# Construction and internal validation of a machine learning model for predicting 30-day readmission after hip surgery

**DOI:** 10.3389/fmed.2026.1872049

**Published:** 2026-08-27

**Authors:** Cuicui Dou, Zhang’an Wang, Keke Dai, Yi Sun, Xiancai Guo, Man Wei, Qiongyan Hu, Bin Yang

**Affiliations:** 1 Faculty of Nursing, Guangxi University of Chinese Medicine, Nanning, Guangxi, China; 2 Department of Anesthesiology, The People’s Hospital of Guangxi Zhuang Autonomous Region, Nanning, Guangxi, China; 3 Department of Health Management, The People’s Hospital of Guangxi Zhuang Autonomous Region, Nanning, Guangxi, China; 4 Department of Obstetrics, The People’s Hospital of Guangxi Zhuang Autonomous Region, Nanning, Guangxi, China; 5 Graduate School of Youjiang Medical University for Nationalities, Baise, Guangxi, China; 6 Department of Joint Surgery and Sports Medicine, The People’s Hospital of Guangxi Zhuang Autonomous Region, Nanning, Guangxi, China

**Keywords:** 30-day readmission, albumin, hip surgery, machine learning, temperature management, XGBoost

## Abstract

**Objective:**

To develop and internally validate a machine learning (ML)-based model for predicting 30-day postoperative readmission after hip surgery using multidimensional perioperative data, and to evaluate its potential clinical utility.

**Methods:**

This single-center retrospective cohort study included 720 patients who underwent hip-related surgery at Guangxi Zhuang Autonomous Region People’s Hospital between 2022 and 2025. Patients were randomly divided into training and test sets at a 7:3 ratio. Demographic characteristics, comorbidities, laboratory variables, anesthesia and temperature-management variables, surgical characteristics, and transfusion-related variables were extracted. Candidate predictors were first selected in the training set using the Boruta algorithm. Eleven base models and one stacking ensemble model were then developed using the selected features. Hyperparameters were optimized using 5-fold cross-validation combined with grid search. Model performance was evaluated using the area under the receiver operating characteristic curve (AUC) and the area under the precision-recall curve (PRAUC), while decision curve analysis (DCA) and SHapley Additive exPlanations (SHAP) were used to assess clinical net benefit and model interpretability.

**Results:**

The overall 30-day readmission rate was 4.3%. In the test set, the XGBoost model achieved an AUC of 0.88 and a PRAUC of 0.53. Across commonly used threshold probabilities, XGBoost provided a higher net benefit than treat-all or treat-none strategies. SHAP analysis identified preoperative albumin, post-anesthesia care unit (PACU) temperature, warming duration, age, and heart failure as the leading predictors of readmission risk.

**Conclusion:**

The XGBoost-based ML model showed potential for predicting 30-day readmission after hip surgery and may provide supportive information for perioperative risk stratification and modifiable management strategies, particularly nutritional optimization and temperature management.

## 1 Introduction

Unplanned readmission within 30 days after hip-related surgery, including total hip arthroplasty (THA) and internal fixation for hip fracture, is an important indicator of health-care quality and perioperative care. It not only increases hospital costs and bed occupancy pressure but is also closely associated with delayed functional recovery and reduced patient satisfaction. Studies based on national or multicenter databases have reported 30-day readmission rates of approximately 2%–8% after elective THA and hip fracture surgery, with leading causes including procedure-related complications, such as prosthetic dislocation, deep infection, and wound problems, as well as cardiovascular and pulmonary medical complications (1).

Traditional studies have generally identified advanced age, a heavy comorbidity burden, such as diabetes, heart failure, and chronic kidney disease, higher American Society of Anesthesiologists (ASA) grade, prolonged operative time, and intraoperative or postoperative complications as important risk factors for early readmission after hip surgery. However, most existing prediction models have been built using multivariable logistic regression, which has a relatively simple model form and may be unable to fully capture nonlinear relationships and interactions among perioperative variables, thereby limiting individualized prediction (2, 3).

With the widespread use of electronic medical records and anesthesia information systems, an increasing number of perioperative variables can be extracted in structured form. These include variables reflecting perioperative temperature management and rewarming strategies, such as preoperative warming, intravenous fluid and irrigation-fluid warming, warming coverage and duration, and body temperature during induction, surgery, and the post-anesthesia care unit (PACU) period. Perioperative hypothermia has been associated with coagulation impairment, increased blood loss and transfusion requirement, altered anesthetic metabolism, and higher risk of surgical-site infection, and it is also common in major orthopedic surgery. Some evidence suggests that hypothermia may be associated with increased 30-day readmission and mortality after hip fracture surgery, although its independent contribution in patients undergoing THA or other hip-related procedures remains unclear (4, 5).

In recent years, machine learning (ML) has attracted increasing attention for predicting readmission after joint arthroplasty. Previous studies have used algorithms such as random forests, gradient boosting trees, and support vector machines to predict 30-day or 90-day readmission after total knee or hip arthroplasty. Some have reported better discrimination than conventional regression models; however, limitations remain, including small sample sizes, restricted feature dimensions, insufficient model generalizability, and limited interpretability. In addition, few studies from single-center Chinese cohorts have systematically incorporated perioperative temperature-management and anesthesia-related rewarming variables while comprehensively comparing multiple ML algorithms and ensemble-learning strategies (6).

Against this background, the present study used a perioperative clinical database of patients who underwent hip-related surgery at Guangxi Zhuang Autonomous Region People’s Hospital from 2022 to 2025. We systematically collected demographic characteristics, comorbidities, preoperative laboratory indicators, anesthesia type, perioperative temperature and rewarming measures, fluid and blood-loss variables, and early postoperative laboratory indicators, and developed ML models to predict unplanned readmission within 30 days after surgery. The aims of this study were: (1) to develop and internally validate individualized readmission-risk prediction models using Boruta feature selection and multiple ML algorithms; (2) to compare the discriminative ability and precision-recall performance of 11 base algorithms and a stacking ensemble model, and to identify the model with the best overall performance; and (3) to evaluate the clinical net benefit of the selected model using decision curve analysis (DCA) and to interpret key predictors using SHapley Additive exPlanations (SHAP), thereby providing quantitative evidence for refined perioperative management and post-discharge follow-up strategies.

## 2 Methods

### 2.1 Study design and participants

This was a single-center retrospective cohort study of consecutive patients who underwent hip-related surgery and completed follow-up at Guangxi Zhuang Autonomous Region People’s Hospital between January 2022 and December 2025. The inclusion criteria were as follows: (1) age 18 years or older; (2) open hip-related surgery, including but not limited to total or hemi-hip arthroplasty, revision surgery, and internal fixation for hip fracture; (3) postoperative follow-up for at least 30 days; and (4) complete perioperative clinical and laboratory data or missingness within an acceptable range. The exclusion criteria were: (1) simple procedures such as non-index admission review surgery or debridement’ with ‘minor procedures unrelated to the index hip operation, such as wound exploration or debridement; (2) perioperative death or transfer to another hospital resulting in missing 30-day readmission information; and (3) severely incomplete records that precluded modeling.

The study was approved by the Ethics Committee of Guangxi Zhuang Autonomous Region People’s Hospital (approval no. KYZC-2024-183). The requirement for informed consent was waived, and all data were de-identified before analysis.

Because this retrospective study included all eligible patients during the prespecified study period, no a priori sample-size calculation was performed. Contemporary prediction-model guidance recommends model-specific sample-size assessment and transparent appraisal of bias rather than reliance on post-hoc power alone (7–9). The final model used five Boruta-selected predictors for 31 readmission events, corresponding to 6.2 events per retained predictor. This ratio is below the commonly cited 10–15 events per predictor heuristic (10) and indicates that the analysis should be considered exploratory, with an appreciable risk of overfitting despite internal validation.

### 2.2 Outcome definition and data collection

#### 2.2.1 Primary outcome

The primary outcome was unplanned readmission within 30 days after surgery (Readmission_30d). It was defined as rehospitalization to any department of the same hospital for any medical reason within 30 days after discharge. Planned readmissions, such as scheduled chemotherapy or rehabilitation, were not counted as outcome events.

#### 2.2.2 Variable extraction and definitions

Data were retrieved from the hospital electronic medical record (EMR) system, anesthesia information management system (AIMS), and laboratory information system (LIS). Four categories of candidate predictors were extracted, as detailed below.

1. Demographic characteristics and baseline comorbidities.

Demographic variables included sex (gender), age, height, weight, body mass index (BMI), and body surface area (BSA). Preoperative comorbidities included hypertension, diabetes, coronary artery disease (CAD), heart failure (heart_failure), stroke or transient ischemic attack (stroke_tia), chronic kidney disease (CKD), chronic obstructive pulmonary disease or asthma (COPD_asthma), thyroid dysfunction, and anemia. Smoking status and alcohol use were also recorded, together with preoperative medication history, including beta-blockers, angiotensin-converting enzyme inhibitors or angiotensin receptor blockers (ACEI/ARBs), antiplatelet agents, and anticoagulants. Overall physical status was assessed using the ASA grade and Charlson comorbidity index (charlson_index).

1. Anesthesia management and perioperative thermal protection.

Anesthesia-related variables included anesthesia type (anesthesia_type; general anesthesia, neuraxial anesthesia, or combined anesthesia) and whether nerve block was performed (nerve_block). Particular attention was paid to perioperative temperature-management details, including preoperative active warming (preop_warming), intraoperative fluid warming (fluid_warming), irrigation-fluid warming (irrigation_warming), and colloid use and volume (colloid_used/volume). For active rewarming measures, warming coverage (warming_coverage; upper body or lower body), warming start time (warming_start_time), and warming duration (warming_duration) were recorded. Temperature variables included preoperative baseline temperature (temp_preop), core temperature at anesthesia induction (temp_induction), 60 min after surgical start (temp_60min), and on admission to the PACU (temp_pacu). Intraoperative hypothermia (intraop_hypothermia) was defined as core temperature below 36.0 °C. Intraoperative shivering and operating-room environmental parameters, including starting temperature (or_temp_start), minimum temperature, and humidity (or_humidity), were also recorded.

1. Surgical characteristics and fluid management.

Surgical variables included operative side (surgery_side), surgical approach (surgical_approach), type of surgery (surgery_type), cement use (cemented), and surgical duration (surgery_duration). Intraoperative circulatory management variables included mean intraoperative mean arterial pressure (map_intraop), minimum mean arterial pressure (map_min), and total opioid dose converted to morphine equivalents (opioid_dose). Fluid-balance variables included estimated blood loss (EBL), postoperative transfusion (postop_transfusion), crystalloid volume, colloid volume, irrigation volume (irrigation_volume), and irrigation temperature (irrigation_temp).

1. Perioperative laboratory variables.

Laboratory variables included key preoperative and early postoperative indicators. Preoperative variables comprised hemoglobin (hb_preop), hematocrit (hct_preop), albumin (albumin_preop), white blood cell count (wbc_preop), and platelet count (platelet_preop). Postoperative hemoglobin levels immediately after surgery (hb_postop) and on postoperative day 1 (POD1; hb_pod1) were collected to evaluate perioperative hemodilution and blood loss.

### 2.3 Data preprocessing and feature selection

The raw data were checked for consistency and logical validity. Extreme outliers were corrected after joint medical-record review where appropriate or otherwise set as missing. Variables with a missing rate of 20% or higher were prespecified for exclusion. No candidate variable met this criterion. Alcohol use was complete, and None, Regular, and Social were treated as valid categories. All retained variables were complete in the analytic dataset; therefore, multiple imputation was neither required nor performed. Continuous variables were retained in their original units and standardized using z-scores before modeling when required by scale-sensitive algorithms. Categorical variables were converted using one-hot encoding. Source-data check: alcohol_use includes 338 None, 70 Regular, and 312 Social observations, with 0 missing values. The former missing-data Supplementary Table S1 must therefore be withdrawn.

Patients were then divided into training and test sets using stratified random sampling at a 7:3 ratio to maintain a comparable 30-day readmission rate between the two sets. To avoid information leakage, feature selection and data standardization were fitted within the training set, and the resulting preprocessing parameters were then applied unchanged to the test set.

Feature selection was performed in the training set using the Boruta algorithm. Boruta repeatedly generates permuted shadow features based on random forests and compares the importance distribution of real features with that of shadow features to identify variables significantly associated with the outcome. All subsequent candidate models were trained, tuned, and compared using the same Boruta-selected feature set, rather than mixing models based on full and selected variable sets.

### 2.4 Machine-learning model development and optimization

#### 2.4.1 Base-model development

Based on the key features selected by Boruta in the training set, 11 ML base models with different architectures were developed and compared using the same feature set, data split, and tuning strategy to capture potential linear and nonlinear relationships among predictors. The included algorithms were grouped into three categories.

Linear and regularized regression models included conventional unpenalized logistic regression, least absolute shrinkage and selection operator (LASSO) regression, ridge regression, and elastic net regression combining L1 and L2 penalties. Tree-based and ensemble-learning algorithms included a single decision tree (DT) and bagging- or boosting-based ensemble models, specifically random forest (RF), extreme gradient boosting (XGBoost), and light gradient boosting machine (LightGBM). Other nonlinear classification algorithms included k-nearest neighbors (KNN), support vector machine (SVM) with a radial basis function kernel, and multilayer perceptron (MLP).

#### 2.4.2 Model training and hyperparameter tuning

To evaluate generalizability and reduce the risk of overfitting, all models were trained on the same Boruta-selected feature set using 5-fold cross-validation combined with grid search in the training set. The primary optimization metric for model performance was the area under the receiver operating characteristic curve (AUC). To ensure a fair comparison, all algorithms were run with the same cross-validation folds and random seed. Given the limited number of positive events, all algorithms were restricted to the same five Boruta-selected predictors. This restriction reduced dimensionality but did not eliminate the risk of overfitting.

#### 2.4.3 Stacking ensemble model

In addition to individual models, a heterogeneous stacking ensemble was constructed to integrate the strengths of different algorithms. The 11 tuned models described above were used as base learners. During training, out-of-fold predicted probabilities were generated from each base learner through cross-validation and used as new input features to construct a meta-dataset. The meta-learner was LASSO logistic regression, which can remove redundant base-model prediction signals through L1 regularization while performing second-level prediction. This strategy aimed to improve ensemble accuracy, reduce overfitting risk, and retain a degree of interpretability.

### 2.5 Performance evaluation and model selection

Model performance was assessed by cross-validation in the training set and finally evaluated in the independent test set. The main discrimination metric was the area under the receiver operating characteristic curve (ROC-AUC). Because 30-day readmission was relatively infrequent, the area under the precision-recall curve (PRAUC) was additionally used as a complementary discrimination metric under class imbalance. Calibration curves were generated in the test set to assess the agreement between predicted probabilities and observed outcomes. To evaluate potential clinical utility, DCA was performed for the final selected model to compare the standardized net benefit of model-guided intervention against treat-all and treat-none strategies across different risk thresholds. To enhance model transparency and clinical interpretability, SHAP values were calculated for the best-performing model to quantify the marginal contribution of each feature to individual predictions, and both global feature-importance rankings and SHAP beeswarm plots were generated. Calibration was additionally quantified using the Brier score, with lower values indicating better overall probabilistic accuracy (11).

### 2.6 Statistical analysis and software

All analyses were performed in R (version 4.4.1) using the caret, Boruta, xgboost, lightgbm, randomForest, e1071, nnet, glmnet, pROC, precrec, and rmda packages.

## 3 Results

### 3.1 Baseline characteristics

A total of 720 eligible patients who underwent hip surgery were included, of whom 31 experienced unplanned readmission within 30 days after surgery, yielding an overall readmission rate of 4.3%.

Baseline characteristics of patients with and without readmission are summarized in Table 1. Compared with patients who were not readmitted, readmitted patients had a worse preoperative comorbidity profile and overall physical status, including a higher Charlson comorbidity index (3.03 ± 0.87 vs. 2.56 ± 1.53, *p* = 0.008), a higher prevalence of heart failure (22.6% vs. 6.1%, *p* = 0.003), and a greater proportion of critically ill patients with ASA class IV (19.4% vs. 10.2%, *p* = 0.005). In laboratory variables, readmitted patients had significantly lower preoperative albumin levels (3.36 ± 0.20 g/dL vs. 3.88 ± 0.40 g/dL, *p* < 0.001).

**Table 1:** Patient demographics and baseline characteristics.

Characteristic	30-day readmission			*p*-value
	Overall *N* = 720	No *N* = 689	Yes *N* = 31	
Surgical duration (min)				0.002^1^
Mean ± SD	106 ± 22	105 ± 22	118 ± 21	
Median (Q1, Q3)	104 (89, 120)	104 (89, 120)	117 (105, 138)	
Min, Max	60, 178	60, 178	74, 164	
Operating-room starting temperature (°C)				0.907^1^
Mean ± SD	21.99 ± 1.16	21.99 ± 1.16	21.96 ± 1.25	
Median (Q1, Q3)	22.02 (20.96, 22.97)	22.04 (20.95, 22.97)	21.73 (20.97, 23.15)	
Min, Max	20.00, 23.99	20.00, 23.99	20.00, 23.88	
Operating-room humidity (%)				0.382^1^
Mean ± SD	40 ± 11	40 ± 11	39 ± 11	
Median (Q1, Q3)	40 (31, 50)	40 (31, 50)	37 (29, 47)	
Min, Max	20, 60	20, 60	21, 60	
Warming start time (min)				0.855^1^
Mean ± SD	9.8 ± 5.9	9.8 ± 5.9	10.0 ± 6.1	
Median (Q1, Q3)	8.6 (5.5, 13.0)	8.6 (5.5, 12.9)	7.7 (5.5, 15.2)	
Min, Max	0.7, 34.8	0.7, 34.8	1.4, 27.3	
Crystalloid volume (mL)				0.631^1^
Mean ± SD	2,225 ± 1,006	2,229 ± 1,004	2,135 ± 1,068	
Median (Q1, Q3)	2,158 (1,352, 3,150)	2,149 (1,358, 3,151)	2,171 (1,042, 2,863)	
Min, Max	501, 3,996	501, 3,996	640, 3,991	
Colloid volume (mL)				0.161^1^
Mean ± SD	125 ± 252	122 ± 248	205 ± 318	
Median (Q1, Q3)	0 (0, 0)	0 (0, 0)	0 (0, 401)	
Min, Max	0, 999	0, 999	0, 979	
Irrigation volume (mL)				0.652^1^
Mean ± SD	1,505 ± 860	1,508 ± 859	1,434 ± 890	
Median (Q1, Q3)	1,543 (826, 2,235)	1,551 (829, 2,231)	1,432 (808, 2,313)	
Min, Max	1, 2,991	1, 2,991	46, 2,977	
Irrigation-fluid temperature (°C)				0.981^1^
Mean ± SD	31.2 ± 7.3	31.2 ± 7.2	31.2 ± 7.5	
Median (Q1, Q3)	36.2 (22.7, 37.2)	36.2 (22.7, 37.2)	36.5 (22.4, 37.0)	
Min, Max	20.0, 38.9	20.0, 38.9	20.0, 38.6	
Estimated blood loss (mL)				0.394^1^
Mean ± SD	286 ± 159	285 ± 159	309 ± 149	
Median (Q1, Q3)	257 (172, 371)	256 (171, 371)	303 (217, 334)	
Min, Max	17, 1,152	17, 1,152	100, 793	
Core temperature at anesthesia induction (°C)				0.095^1^
Mean ± SD	36.26 ± 0.30	36.26 ± 0.30	36.17 ± 0.29	
Median (Q1, Q3)	36.26 (36.05, 36.46)	36.26 (36.06, 36.46)	36.21 (35.93, 36.39)	
Min, Max	35.27, 37.17	35.27, 37.17	35.62, 36.67	
Core temperature 60 min after surgical start (°C)				0.052^1^
Mean ± SD	35.47 ± 0.38	35.47 ± 0.38	35.35 ± 0.34	
Median (Q1, Q3)	35.47 (35.20, 35.71)	35.47 (35.20, 35.71)	35.38 (35.03, 35.62)	
Min, Max	34.32, 36.77	34.32, 36.77	34.58, 35.85	
Mean intraoperative arterial pressure (mmHg)				0.316^1^
Mean ± SD	81 ± 10	81 ± 10	79 ± 11	
Median (Q1, Q3)	81 (73, 87)	81 (73, 87)	82 (70, 87)	
Min, Max	56, 107	57, 107	56, 103	
Minimum mean arterial pressure (mmHg)				0.484^1^
Mean ± SD	68 ± 11	68 ± 11	66 ± 12	
Median (Q1, Q3)	68 (61, 76)	68 (61, 75)	65 (55, 77)	
Min, Max	39, 96	39, 96	47, 95	
Core temperature at PACU admission (degrees C)				0.214^1^
Mean ± SD	36.41 ± 0.37	36.41 ± 0.37	36.31 ± 0.42	
Median (Q1, Q3)	36.42 (36.15, 36.66)	36.43 (36.15, 36.66)	36.28 (36.06, 36.65)	
Min, Max	35.51, 37.49	35.51, 37.49	35.61, 37.17	
Immediate postoperative hemoglobin (g/dL)				0.901^1^
Mean ± SD	11.60 ± 1.52	11.61 ± 1.52	11.57 ± 1.45	
Median (Q1, Q3)	11.60 (10.47, 12.60)	11.60 (10.47, 12.60)	11.69 (10.59, 12.62)	
Min, Max	7.46, 15.82	7.46, 15.82	8.93, 14.43	
Hemoglobin on postoperative day 1 (g/dL)				0.525^1^
Mean ± SD	10.80 ± 1.58	10.79 ± 1.58	10.96 ± 1.44	
Median (Q1, Q3)	10.80 (9.74, 11.84)	10.80 (9.72, 11.84)	10.76 (10.00, 11.96)	
Min, Max	6.65, 15.13	6.65, 15.13	7.07, 13.76	
Preoperative hemoglobin (g/dL)				0.654^1^
Mean ± SD	13.62 ± 1.45	13.62 ± 1.45	13.74 ± 1.41	
Median (Q1, Q3)	13.59 (12.55, 14.67)	13.59 (12.55, 14.67)	13.60 (12.64, 15.01)	
Min, Max	10.14, 17.73	10.14, 17.73	10.79, 17.12	
Preoperative hematocrit (%)				0.637^1^
Mean ± SD	40.9 ± 4.4	40.9 ± 4.4	41.2 ± 4.3	
Median (Q1, Q3)	40.8 (37.7, 43.9)	40.8 (37.7, 43.9)	40.4 (37.8, 45.0)	
Min, Max	29.5, 53.4	29.5, 53.4	33.1, 50.2	
Preoperative albumin (g/dL)				<0.001^1^
Mean ± SD	3.86 ± 0.41	3.88 ± 0.40	3.36 ± 0.20	
Median (Q1, Q3)	3.86 (3.56, 4.15)	3.89 (3.60, 4.16)	3.34 (3.25, 3.47)	
Min, Max	2.51, 4.91	2.51, 4.91	3.07, 3.90	
Preoperative white blood cell count (x10^9^/L)				0.899^1^
Mean ± SD	7.13 ± 1.89	7.13 ± 1.88	7.18 ± 2.26	
Median (Q1, Q3)	7.04 (5.84, 8.42)	7.04 (5.88, 8.43)	6.24 (5.24, 8.38)	
Min, Max	3.09, 12.61	3.09, 12.61	4.47, 11.82	
Preoperative platelet count (x10^9^/L)				0.132^1^
Mean ± SD	243 ± 59	242 ± 59	258 ± 56	
Median (Q1, Q3)	240 (201, 282)	239 (201, 281)	266 (219, 292)	
Min, Max	105, 469	105, 469	114, 358	
Preoperative core temperature (°C)				0.311^1^
Mean ± SD	36.60 ± 0.29	36.60 ± 0.29	36.55 ± 0.29	
Median (Q1, Q3)	36.61 (36.41, 36.80)	36.61 (36.41, 36.80)	36.62 (36.29, 36.82)	
Min, Max	35.57, 37.47	35.57, 37.47	36.02, 37.06	
Age (years)				0.501^1^
Mean ± SD	74.5 ± 5.3	74.5 ± 5.3	75.2 ± 6.1	
Median (Q1, Q3)	74.0 (70.0, 78.0)	74.0 (70.0, 78.0)	73.0 (72.0, 77.0)	
Min, Max	65.0, 94.0	65.0, 94.0	67.0, 93.0	
Height (m)				0.423^1^
Mean ± SD	1.58 ± 0.06	1.58 ± 0.06	1.59 ± 0.05	
Median (Q1, Q3)	1.58 (1.54, 1.62)	1.58 (1.54, 1.62)	1.60 (1.54, 1.64)	
Min, Max	1.45, 1.75	1.45, 1.75	1.47, 1.68	
Body mass index (kg/m^2^)				0.080^1^
Mean ± SD	29.1 ± 4.8	29.1 ± 4.8	27.6 ± 4.5	
Median (Q1, Q3)	29.0 (25.6, 32.5)	29.0 (25.7, 32.6)	28.1 (22.8, 30.3)	
Min, Max	18.2, 43.0	18.2, 43.0	19.6, 38.2	
Weight (kg)				0.257^1^
Mean ± SD	73 ± 13	73 ± 13	70 ± 13	
Median (Q1, Q3)	73 (63, 82)	73 (63, 82)	71 (58, 77)	
Min, Max	43, 113	43, 113	45, 101	
Body surface areaBody surface area (m^2^)				0.382^1^
Mean ± SD	1.78 ± 0.18	1.78 ± 0.18	1.75 ± 0.19	
Median (Q1, Q3)	1.78 (1.65, 1.89)	1.78 (1.65, 1.90)	1.78 (1.62, 1.86)	
Min, Max	1.35, 2.30	1.35, 2.30	1.37, 2.14	
Warming duration (min)				0.367^1^
Mean ± SD	22.3 ± 6.0	22.3 ± 5.9	23.8 ± 9.1	
Median (Q1, Q3)	20.0 (20.0, 20.0)	20.0 (20.0, 20.0)	20.0 (20.0, 21.4)	
Min, Max	20.0, 60.0	20.0, 60.0	20.0, 50.2	
Total opioid dose (mg morphine equivalents)				0.955^1^
Mean ± SD	20 ± 13	20 ± 14	19 ± 11	
Median (Q1, Q3)	16 (11, 25)	16 (11, 25)	19 (11, 24)	
Min, Max	2, 124	2, 124	4, 47	
Charlson comorbidity index				0.008^1^
Mean ± SD	2.58 ± 1.51	2.56 ± 1.53	3.03 ± 0.87	
Median (Q1, Q3)	3.00 (1.00, 4.00)	3.00 (1.00, 4.00)	3.00 (2.00, 4.00)	
Min, Max	0.00, 9.00	0.00, 9.00	1.00, 5.00	
Intraoperative shivering, *n* (%)				0.112^2^
No	649 (90.1%)	624 (90.6%)	25 (80.6%)	
Yes	71 (9.9%)	65 (9.4%)	6 (19.4%)	
Sex, *n* (%)				0.351^3^
Female	406 (56.4%)	386 (56.0%)	20 (64.5%)	
Male	314 (43.6%)	303 (44.0%)	11 (35.5%)	
Postoperative transfusion, *n* (%)				<0.001^2^
No	685 (95.1%)	661 (95.9%)	24 (77.4%)	
Yes	35 (4.9%)	28 (4.1%)	7 (22.6%)	
Intraoperative hypothermia, *n* (%)				0.002^3^
No	442 (61.4%)	431 (62.6%)	11 (35.5%)	
Yes	278 (38.6%)	258 (37.4%)	20 (64.5%)	
Diabetes, *n* (%)				0.385^3^
No	557 (77.4%)	535 (77.6%)	22 (71.0%)	
Yes	163 (22.6%)	154 (22.4%)	9 (29.0%)	
Hypertension, *n* (%)				0.562^3^
No	244 (33.9%)	232 (33.7%)	12 (38.7%)	
Yes	476 (66.1%)	457 (66.3%)	19 (61.3%)	
Coronary artery disease, *n* (%)				0.788^2^
No	621 (86.3%)	593 (86.1%)	28 (90.3%)	
Yes	99 (13.8%)	96 (13.9%)	3 (9.7%)	
Heart failure, *n* (%)				0.003^2^
No	671 (93.2%)	647 (93.9%)	24 (77.4%)	
Yes	49 (6.8%)	42 (6.1%)	7 (22.6%)	
Stroke or transient ischemic attack, *n* (%)				>0.999^2^
No	665 (92.4%)	636 (92.3%)	29 (93.5%)	
Yes	55 (7.6%)	53 (7.7%)	2 (6.5%)	
Chronic obstructive pulmonary disease or asthma, *n* (%)				>0.999^2^
No	623 (86.5%)	596 (86.5%)	27 (87.1%)	
Yes	97 (13.5%)	93 (13.5%)	4 (12.9%)	
Chronic kidney disease, *n* (%)				>0.999^2^
No	642 (89.2%)	614 (89.1%)	28 (90.3%)	
Yes	78 (10.8%)	75 (10.9%)	3 (9.7%)	
Thyroid dysfunction, *n* (%)				>0.999^2^
No	654 (90.8%)	625 (90.7%)	29 (93.5%)	
Yes	66 (9.2%)	64 (9.3%)	2 (6.5%)	
Anemia, *n* (%)				0.783^3^
No	549 (76.3%)	526 (76.3%)	23 (74.2%)	
Yes	171 (23.8%)	163 (23.7%)	8 (25.8%)	
Beta-blocker use, *n* (%)				0.961^3^
No	560 (77.8%)	536 (77.8%)	24 (77.4%)	
Yes	160 (22.2%)	153 (22.2%)	7 (22.6%)	
ACE inhibitor or ARB use, *n* (%)				0.290^3^
No	437 (60.7%)	421 (61.1%)	16 (51.6%)	
Yes	283 (39.3%)	268 (38.9%)	15 (48.4%)	
Antiplatelet, *n* (%)				0.500^3^
No	544 (75.6%)	519 (75.3%)	25 (80.6%)	
Yes	176 (24.4%)	170 (24.7%)	6 (19.4%)	
Anticoagulant, *n* (%)				0.527^2^
No	651 (90.4%)	624 (90.6%)	27 (87.1%)	
Yes	69 (9.6%)	65 (9.4%)	4 (12.9%)	
Preoperative active warming, *n* (%)				0.887^3^
No	450 (62.5%)	431 (62.6%)	19 (61.3%)	
Yes	270 (37.5%)	258 (37.4%)	12 (38.7%)	
Intraoperative fluid warming, *n* (%)				0.112^3^
No	190 (26.4%)	178 (25.8%)	12 (38.7%)	
Yes	530 (73.6%)	511 (74.2%)	19 (61.3%)	
Irrigation-fluid warming, *n* (%)				0.932^3^
No	284 (39.4%)	272 (39.5%)	12 (38.7%)	
Yes	436 (60.6%)	417 (60.5%)	19 (61.3%)	
Colloid use, *n* (%)				0.144^3^
No	544 (75.6%)	524 (76.1%)	20 (64.5%)	
Yes	176 (24.4%)	165 (23.9%)	11 (35.5%)	
Nerve block, *n* (%)				0.722^3^
No	440 (61.1%)	422 (61.2%)	18 (58.1%)	
Yes	280 (38.9%)	267 (38.8%)	13 (41.9%)	
ASA physical status grade, *n* (%)				0.005^2^
I	11 (1.5%)	8 (1.2%)	3 (9.7%)	
II	261 (36.3%)	250 (36.3%)	11 (35.5%)	
III	372 (51.7%)	361 (52.4%)	11 (35.5%)	
IV	76 (10.6%)	70 (10.2%)	6 (19.4%)	
Smoking status, *n* (%)				0.356^2^
Current	83 (11.5%)	81 (11.8%)	2 (6.5%)	
Former	281 (39.0%)	265 (38.5%)	16 (51.6%)	
Never	356 (49.4%)	343 (49.8%)	13 (41.9%)	
Alcohol use, *n* (%)				0.660^2^
None	338 (46.9%)	325 (47.2%)	13 (41.9%)	
Regular	70 (9.7%)	68 (9.9%)	2 (6.5%)	
Social	312 (43.3%)	296 (43.0%)	16 (51.6%)	
Anesthesia type, *n* (%)				0.515^3^
General	296 (41.1%)	285 (41.4%)	11 (35.5%)	
Neuraxial	424 (58.9%)	404 (58.6%)	20 (64.5%)	
Operative side, *n* (%)				0.547^3^
Left	340 (47.2%)	327 (47.5%)	13 (41.9%)	
Right	380 (52.8%)	362 (52.5%)	18 (58.1%)	
Surgical approach, *n* (%)				0.079^2^
Anterior	230 (31.9%)	218 (31.6%)	12 (38.7%)	
Lateral	88 (12.2%)	81 (11.8%)	7 (22.6%)	
Posterior	402 (55.8%)	390 (56.6%)	12 (38.7%)	
Cement use, *n* (%)				0.348^2^
No	653 (90.7%)	623 (90.4%)	30 (96.8%)	
Yes	67 (9.3%)	66 (9.6%)	1 (3.2%)	
Surgery type, *n* (%)				0.050^2^
Primary	700 (97.2%)	672 (97.5%)	28 (90.3%)	
Revision	20 (2.8%)	17 (2.5%)	3 (9.7%)	
Warming coverage, *n* (%)				0.123^3^
Full Body	355 (49.3%)	339 (49.2%)	16 (51.6%)	
Lower Body	223 (31.0%)	210 (30.5%)	13 (41.9%)	
Upper Body	142 (19.7%)	140 (20.3%)	2 (6.5%)	

1 Welch Two Sample *t*-test.

2 Fisher’s exact test.

3 Pearson’s Chi-squared test. ACE, angiotensin-converting enzyme; ARB, angiotensin receptor blocker; ASA, American Society of Anesthesiologists; BMI, body mass index; BSA, body surface area; CAD, coronary artery disease; CKD, chronic kidney disease; COPD, chronic obstructive pulmonary disease; EBL, estimated blood loss; MAP, mean arterial pressure; n, number of patients in a category; N, total number of patients; PACU, post-anesthesia care unit; POD1, postoperative day 1; Q1, first quartile; Q3, third quartile; SD, standard deviation; TIA, transient ischemic attack; WBC, white blood cell.

Regarding perioperative management and surgical variables, surgical duration was significantly longer in the readmission group than in the non-readmission group (118 ± 21 min vs. 105 ± 22 min, *p* = 0.002). Notably, the incidence of intraoperative hypothermia (intraop_hypothermia) was significantly higher among readmitted patients (64.5% vs. 37.4%, *p* = 0.002), and postoperative transfusion (postop_transfusion) was more frequent (22.6% vs. 4.1%, *p* < 0.001).

No statistically significant between-group differences were observed in age (*p* = 0.501), sex (*p* = 0.351), BMI (*p* = 0.080), history of diabetes (*p* = 0.385), estimated blood loss (EBL; *p* = 0.394), preoperative hemoglobin (p = 0.654), or postoperative shivering (*p* = 0.112). Neuraxial anesthesia was the most common anesthesia type (58.9%), and the posterior approach was the most common surgical approach (55.8%), with balanced distributions between groups.

### 3.2 Boruta feature selection

**Figure 1:**
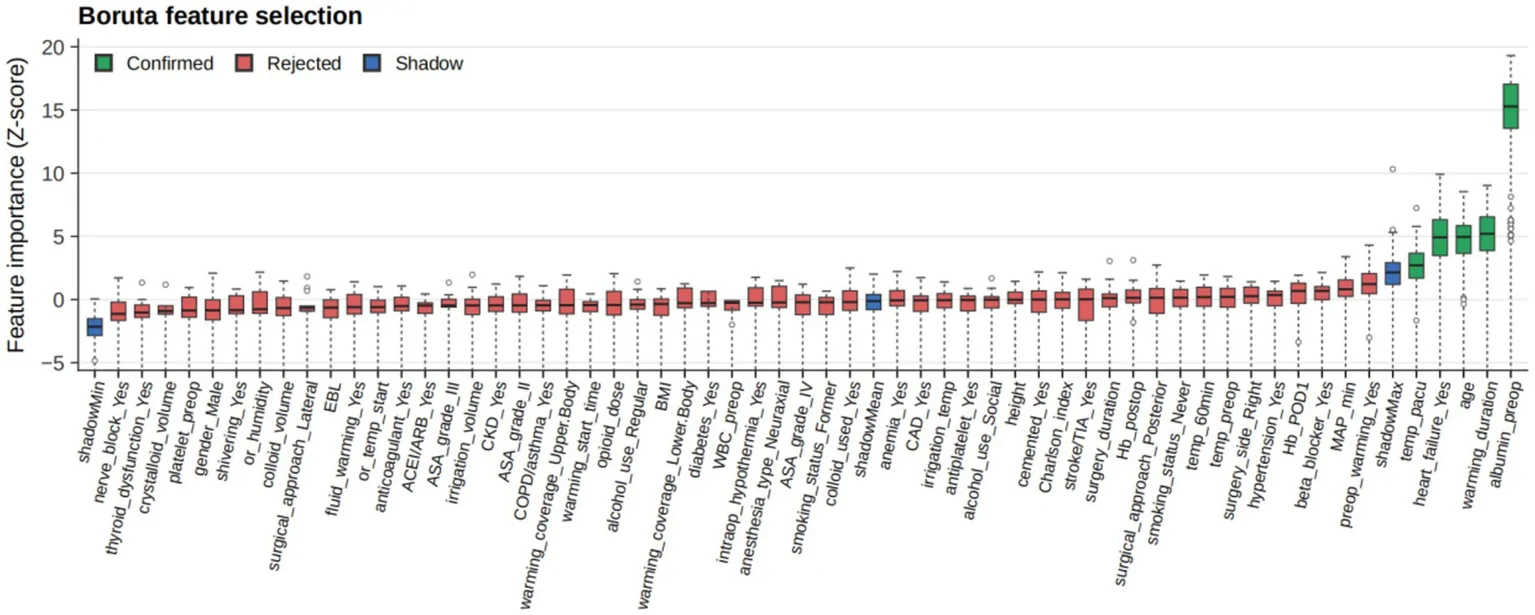
Feature importance ranking for predicting 30-day readmission after hip surgery based on the Boruta algorithm. The *y*-axis represents the feature-importance *Z*-score. The five green boxplots indicate confirmed key features, including preoperative albumin, warming duration, age, PACU temperature, and heart failure, which were used in subsequent model development, tuning, and performance comparison. Red boxplots indicate rejected features with importance scores lower than random noise represented by ShadowMax. Blue boxplots indicate the threshold values of shadow features.

The Boruta algorithm was applied to all candidate variables in the training set. By comparing feature-importance Z-scores with randomly generated shadow features, Boruta identified variables significantly associated with 30-day postoperative readmission. As shown in Figure 1, after multiple iterations, only five of more than 60 candidate variables were confirmed as important for predicting 30-day readmission (green boxplots). In descending order of importance, these variables were preoperative albumin (albumin_preop), which had the highest importance score and represented the strongest predictor, warming duration (warming_duration), age, PACU admission temperature (temp_pacu), and heart failure (heart_failure_Yes). The remaining variables, including conventional ASA grade, Charlson comorbidity index, preoperative comorbidities, and most hemodynamic indicators, had Z-scores below or close to ShadowMax (blue boxplots) and were classified as rejected features (red boxplots). This indicates that their predictive contribution did not exceed random noise in the present dataset, and they were therefore not included in final model development.

### 3.3 Model performance evaluation

#### 3.3.1 Cross-validation stage

**Figure 2:**
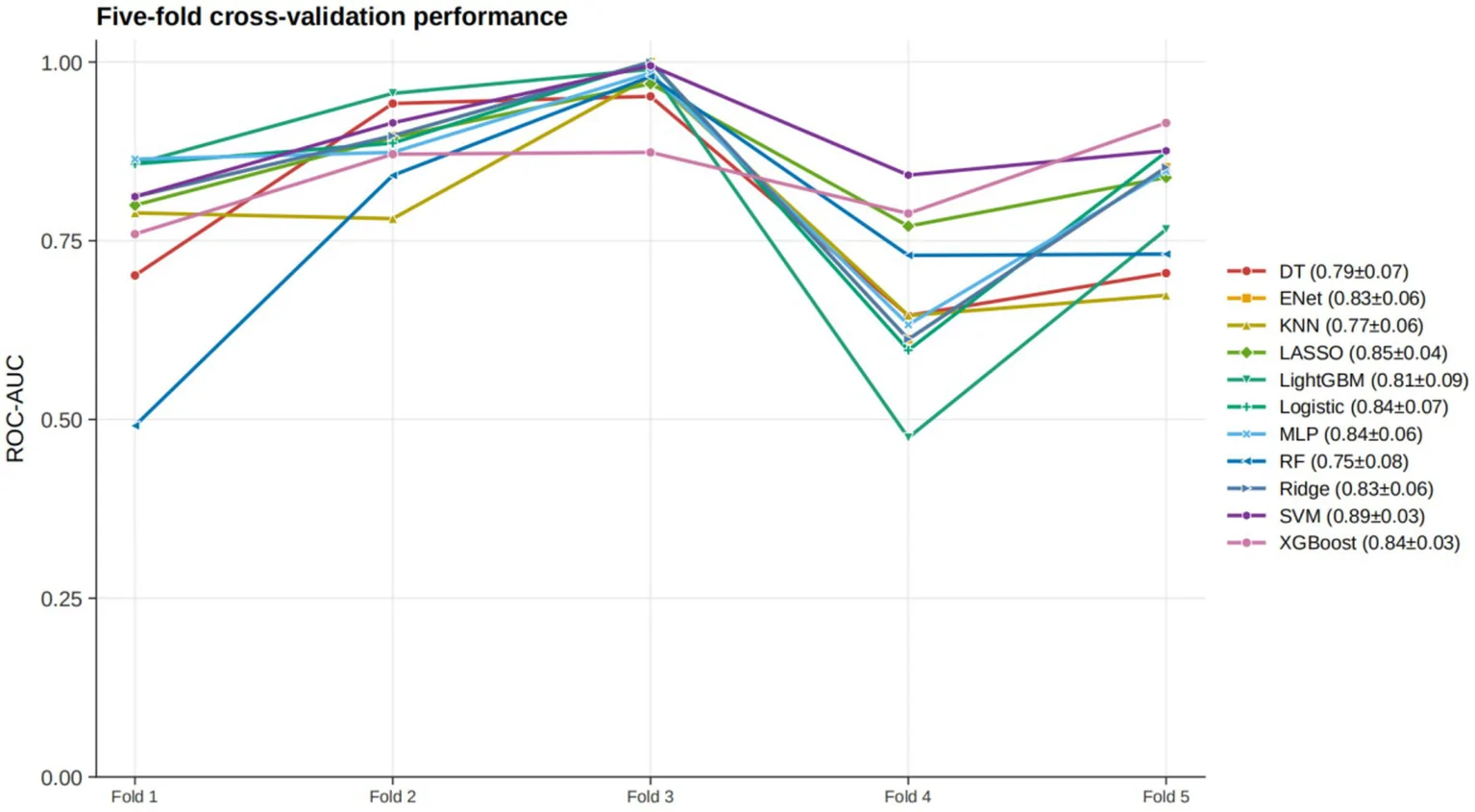
Comparison of ROC-AUC performance among 11 ML models during 5-fold cross-validation in the training set. The line chart shows AUC trends for each model across folds 1–5. Values in the legend are presented as mean AUC ± standard deviation (SD). SVM, support vector machine; MLP, multilayer perceptron; DT, decision tree; RF, random forest; KNN, k-nearest neighbors.

During 5-fold cross-validation in the training set, ROC-AUC trends and mean performance across the 11 ML algorithms are shown in Figure 2. Hyperparameter search spaces and final selected parameters for each model are provided in Supplementary Table S1. Overall, most models showed good discrimination. SVM achieved the highest mean performance, with an AUC of 0.89 ± 0.03 and stable performance across folds, as reflected by the small standard deviation. Among linear models, LASSO regression ranked second, with an AUC of 0.85 ± 0.04. Algorithms in the second tier included logistic regression (0.84 ± 0.07), MLP (0.84 ± 0.06), and XGBoost (0.84 ± 0.03). Notably, XGBoost and SVM both had a standard deviation of 0.03, indicating robust generalization across different data subsets. In contrast, RF (0.75 ± 0.08) and KNN (0.77 ± 0.06) performed relatively weakly during cross-validation, and RF showed greater performance variability (SD = 0.08).

#### 3.3.2 Training-set and test-set validation

**Figure 3:**
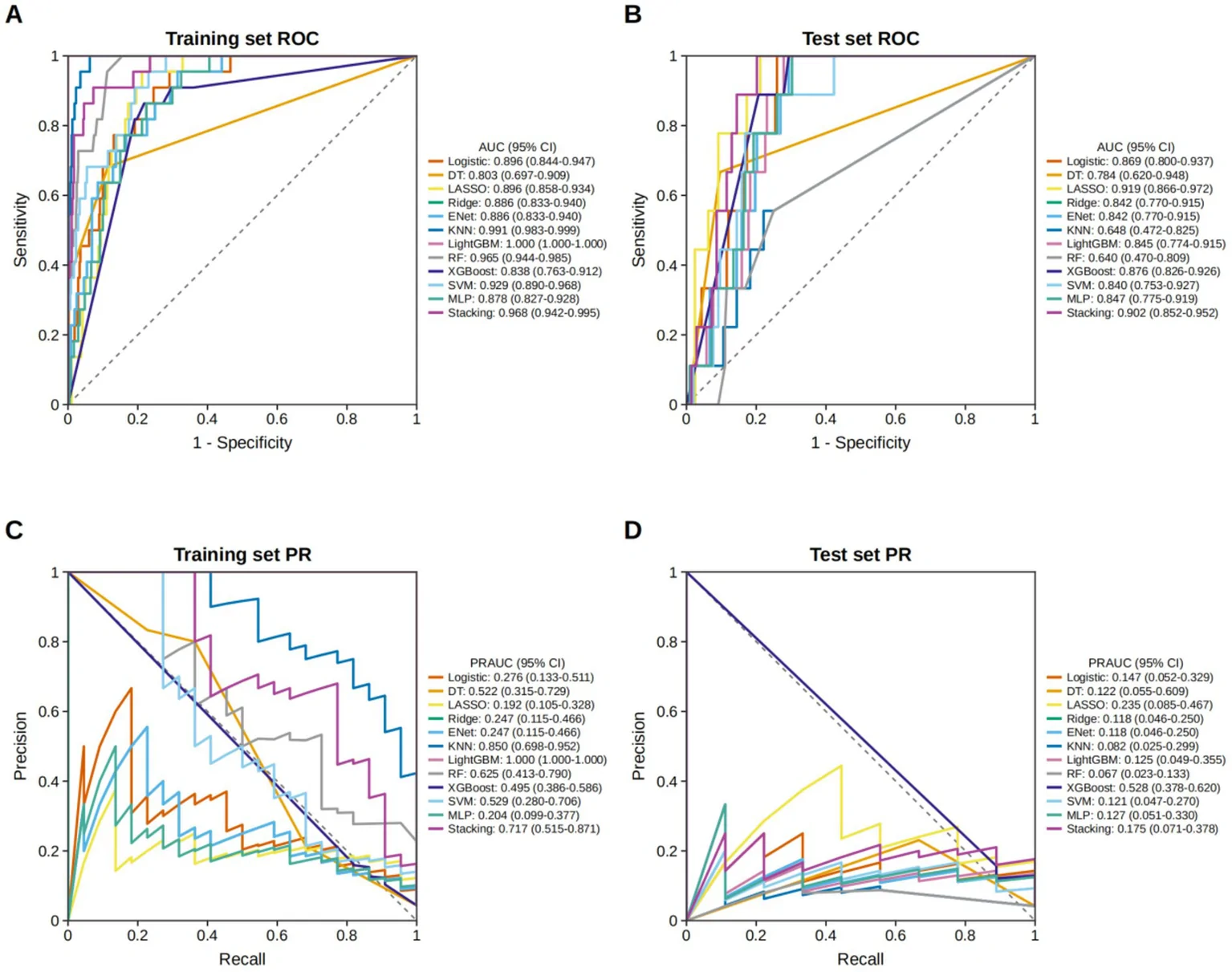
Comparison of receiver operating characteristic (ROC) and precision-recall (PR) curves among 12 ML models in the training and test sets. **(A)** Training-set ROC curves: LightGBM, KNN, and RF exhibited extremely high AUCs (>0.96), suggesting potential overfitting. **(B)** Test-set ROC curves: LASSO, stacking, and XGBoost showed favorable generalization performance (AUC > 0.87), whereas RF and KNN declined markedly. **(C)** Training-set PR curves: Most models showed good identification of positive samples in the training data. **(D)** Test-set PR curves: XGBoost achieved a relatively high PRAUC (0.5280) in independent validation, suggesting a degree of ability to identify positive events under class imbalance. Values in the legends represent AUC or PRAUC, with 95% confidence intervals where applicable.

To evaluate model fit and generalizability, model performance was compared between the training set and the independent test set. Four panels show the training-set receiver operating characteristic (ROC) curves (Figure 3A), test-set ROC curves (Figure 3B), training-set precision-recall (PR) curves (Figure 3C), and test-set PR curves (Figure 3D).

In the training set, most nonlinear models showed very high apparent fitting ability. In ROC analysis (Figure 3A), LightGBM achieved perfect classification (AUC = 1.0000), followed by KNN (AUC = 0.9909) and RF (AUC = 0.9648), suggesting strong sample memorization. The stacking ensemble also achieved a high AUC of 0.9683. XGBoost showed a more moderate training-set AUC of 0.8376. In training-set PR analysis (Figure 3C), LightGBM again achieved perfect prediction (PRAUC = 1.0000), while KNN (PRAUC = 0.8495) and stacking (PRAUC = 0.7165) also performed well. The training-set PRAUC of XGBoost was 0.4955.

When applied to the independent test set (Figures 3B,D), model performance diverged substantially, revealing overfitting in some algorithms. Although RF and KNN performed well in the training set, their test-set AUCs decreased markedly to 0.6397 and 0.6485, respectively, indicating poor generalization due to overfitting to training noise. In contrast, linear models were relatively stable in the test set, with LASSO (AUC = 0.9193) and stacking (AUC = 0.9022) ranking among the top models. XGBoost achieved a test-set AUC of 0.8763 and remained at a high overall level. In PR-curve analysis, which is more informative for imbalanced outcomes, XGBoost achieved a test-set PRAUC of 0.5280, exceeding LASSO (0.2355) and stacking (0.1749), suggesting a relative advantage in identifying positive readmission events.

Taken together, Figure 3 shows that LightGBM and RF achieved near-perfect performance in the training set but substantially lower performance in the test set, indicating an evident risk of overfitting. By contrast, XGBoost showed a more balanced performance between the training and test sets and was therefore selected as the candidate model for subsequent interpretability and clinical net-benefit analyses. However, although XGBoost achieved relatively favorable overall performance in the internal test set, its absolute PRAUC remained moderate, indicating that the model is more suitable for supportive risk stratification than for independent clinical decision-making.

### 3.4 Decision-curve and calibration analyses

**Figure 4:**
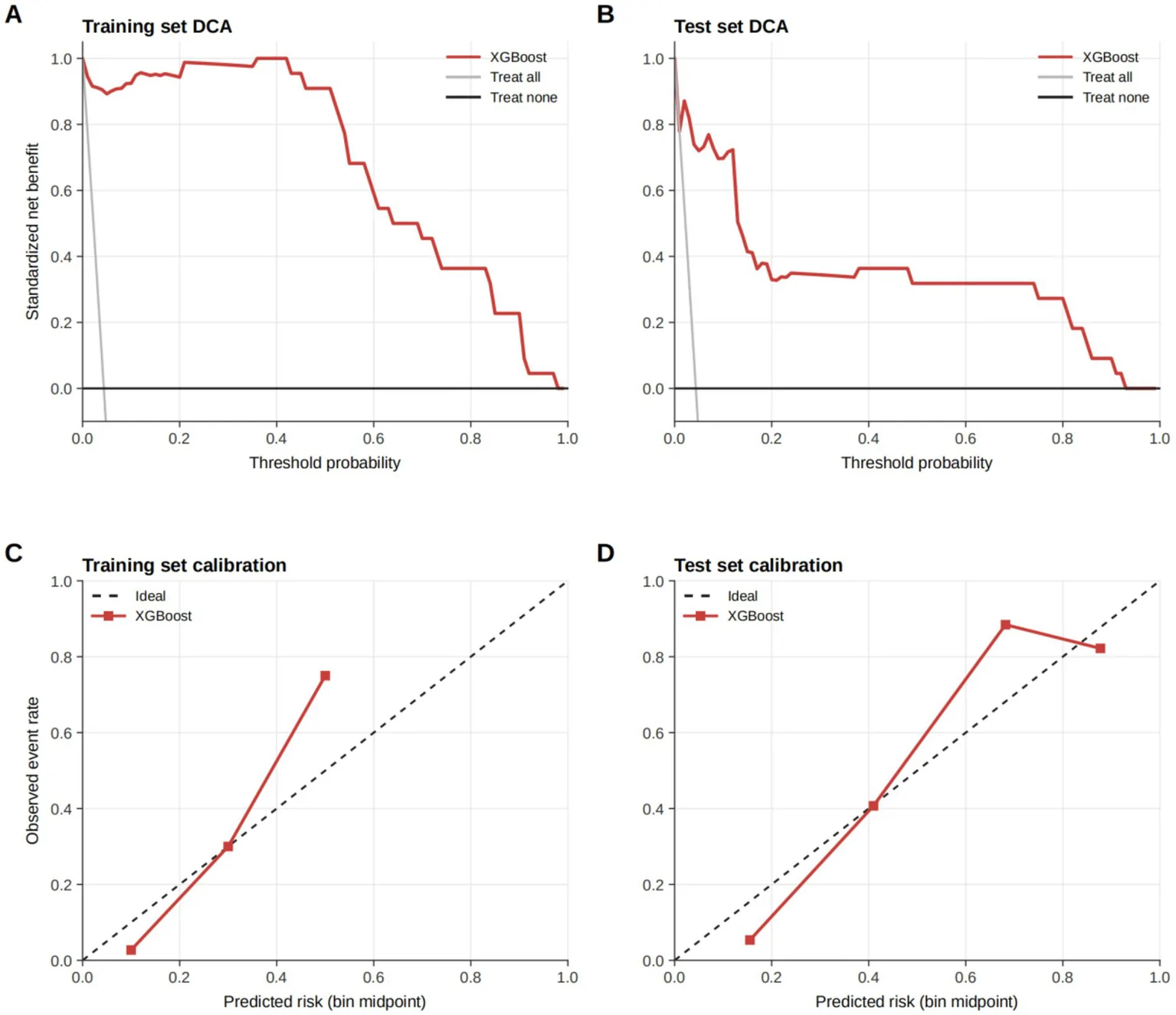
Clinical net benefit and calibration performance of the final XGBoost model in the training and test sets **(A)** Decision curve analysis (DCA) in the training set; **(B)** DCA in the test set; **(C)** Calibration curve in the training set; and **(D)** Calibration curve in the test set. In **A** and **B**, the red curve represents the standardized net benefit of XGBoost, the grey curve represents the treat-all strategy, and the black horizontal line represents the treat-none strategy. In **C** and **D**, the dashed line indicates ideal calibration, and the red solid line shows the agreement between predicted and observed probabilities. The Brier scores were 0.0396 for the training set and 0.0375 for the test set.

To comprehensively assess the clinical utility of the selected model and the agreement between predicted probabilities and observed outcomes, DCA and calibration curves were generated for the XGBoost model in both the training and test sets (Figure 4). Figures 4A,B show DCA results in the training and test sets, respectively. The red curve represents the standardized net benefit of XGBoost, the grey curve represents the treat-all strategy, and the black horizontal line represents the treat-none strategy. Because 30-day readmission after hip surgery was a low-frequency event, the net benefit of the treat-all strategy decreased rapidly as the threshold probability increased and approached or fell below zero at relatively low thresholds. In contrast, XGBoost achieved a relatively higher standardized net benefit across a broad range of threshold probabilities in both the training and test sets. This suggests that model-guided intensified perioperative management may provide greater clinical decision benefit than treat-all or treat-none strategies. The net-benefit curve in the test set remained generally favorable, although it fluctuated more visibly, consistent with the low event rate and limited sample size.

Figures 4C,D show calibration curves in the training and test sets, respectively. Overall, predicted probabilities were reasonably consistent with the observed probability of readmission, indicating that XGBoost had some ability to estimate absolute risk. The test-set calibration curve showed deviations in some risk ranges, suggesting that calibration performance requires further validation in larger and external datasets. Overall, XGBoost demonstrated good discrimination, a degree of clinical net benefit, and internally consistent risk estimation; however, the findings should be interpreted cautiously given the single-center design and low event rate. The Brier scores were 0.0396 in the training set and 0.0375 in the test set.

### 3.5 SHAP interpretability analysis

**Figure 5:**
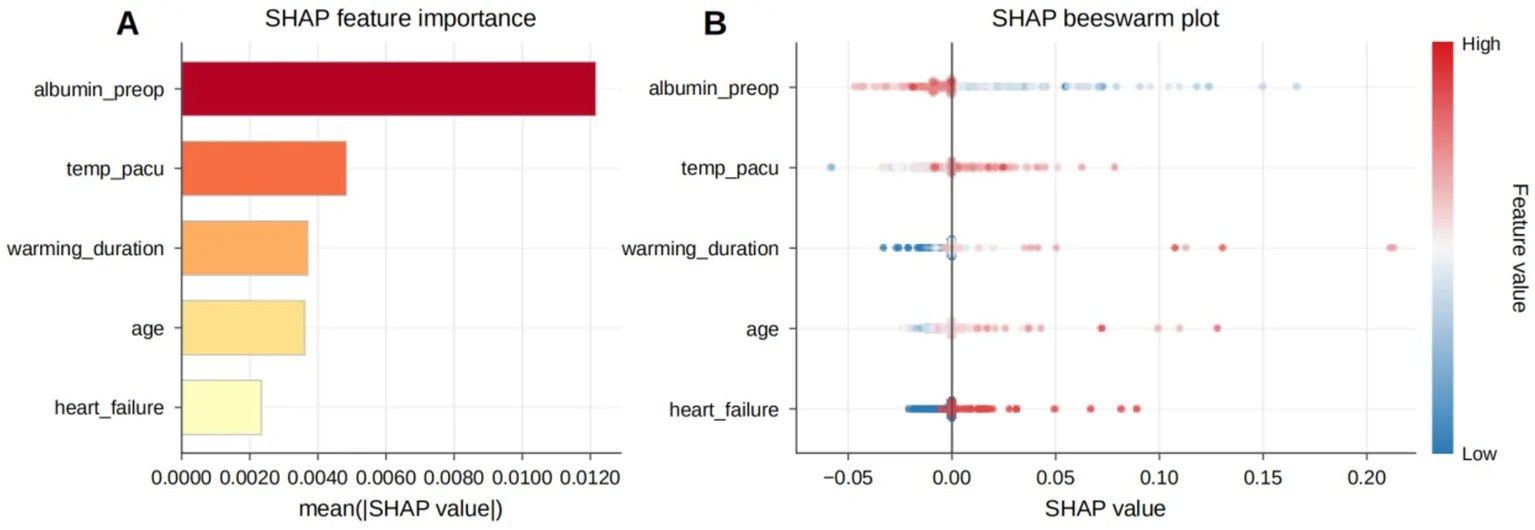
SHAP interpretation of the final XGBoost model: **(A)** Global feature importance ranked by mean absolute SHAP values. **(B)** SHAP beeswarm plot showing the direction and magnitude of each feature’s contribution to the predicted risk of 30-day readmission. Each dot represents one patient; red indicates higher feature values and blue indicates lower feature values. Positive SHAP values indicate increased predicted risk. Preoperative albumin, PACU temperature, warming duration, age, and heart failure were the most influential predictors.

To reveal the decision logic of the XGBoost black-box model, SHAP was used to visualize feature contributions and directions of effect (Figure 5). Figure 5A shows the global feature-importance ranking based on mean absolute SHAP values. Preoperative albumin (albumin_preop) contributed the most to model output and was the most important predictor. The top five features were preoperative albumin, PACU admission temperature, intraoperative warming duration, age, and heart failure. These findings suggest that baseline nutritional status and perioperative temperature management contributed more strongly to readmission risk than conventional demographic variables. The SHAP beeswarm plot (Figure 5B) further shows the relationship between individual feature values and predicted readmission risk. Each point represents one patient, and color indicates the feature value, with red denoting high values and blue denoting low values. A SHAP value greater than 0 indicates increased readmission risk, whereas a value below 0 indicates reduced risk. For preoperative albumin, blue points representing lower albumin levels were mainly distributed to the right of the central axis (SHAP > 0), indicating that lower albumin was associated with higher predicted 30-day readmission risk. Temperature-management variables showed a clear negative association with risk: lower PACU temperature (blue points) was associated with higher risk, and shorter warming duration was also distributed on the risk-increasing side. For age and heart failure, red points, representing older age or the presence of heart failure, were mainly located on the right side, indicating their positive contribution to the model’s predicted risk.

## 4 Discussion

In this single-center cohort of 720 patients who underwent hip-related surgery at Guangxi Zhuang Autonomous Region People’s Hospital, we developed and internally validated an ML model for predicting unplanned readmission within 30 days after surgery. Through Boruta feature selection, multi-algorithm comparison, and stacking ensemble modeling, we found that several ML algorithms showed favorable discrimination. XGBoost achieved a balanced ROC-AUC (0.8763) and PRAUC (0.5280) in the independent test set and showed better identification of high-risk patients than other models under class imbalance. DCA suggested that the model provided meaningful net benefit across a range of clinically acceptable thresholds. SHAP analysis further identified key contributors to readmission risk, including low preoperative albumin, heart failure, older age, low PACU temperature, and insufficient intraoperative warming duration.

Many previous studies have used data from the National Surgical Quality Improvement Program (NSQIP) or other large databases and applied multivariable logistic regression to identify factors associated with 30-day readmission after hip arthroplasty or hip fracture surgery. These studies reported advanced age, higher ASA grade, diabetes, heart failure, and prolonged operative time as key risk factors. Ali et al. analyzed more than 510,000 THA procedures in the UK National Health Service and found that age, comorbidity burden, and longer length of stay were significantly associated with all-cause readmission; they also suggested that risk factors for surgery-related readmission differ from those for medical readmission. Our findings are highly consistent with this literature, as age and heart failure were important predictors in both Boruta and SHAP analyses (1, 2, 5). More recent arthroplasty cohorts have likewise identified patient and procedural factors associated with early readmission, while externally validated ML studies have shown that transportable performance cannot be assumed from internal results alone (12–14).

Among more than 60 candidate variables, Boruta ultimately identified only five features significantly associated with 30-day postoperative readmission. This result does not imply that other perioperative variables are clinically unimportant. Rather, it may reflect the limited number of outcome events in the present study: only 31 of 720 patients experienced 30-day readmission. The low event count may limit the stability of feature selection and increase uncertainty in model-performance estimates. At the same time, Boruta is a relatively conservative all-relevant feature-selection method that tends to retain features with stable predictive contributions above random noise. Therefore, our results should be interpreted as identifying a small set of stable key signals under the current sample and event structure, rather than demonstrating that other variables have no predictive value. With five retained predictors, the effective ratio was 6.2 readmission events per predictor, below the conventional 10–15 events-per-predictor heuristic. Thus, the available sample was limited for definitive prediction-model development, and the reported discrimination and feature rankings may be optimistic or unstable.

From a methodological perspective, previous studies have begun to explore ML for predicting readmission after joint arthroplasty. For example, Mohammadi et al. used ML models to predict 30-day readmission after knee or hip arthroplasty and reported that gradient boosting and random forests outperformed conventional regression in terms of AUC. Wu et al. applied ML to predict 30-day readmission after THA and hemiarthroplasty and achieved acceptable discrimination, but the sample size was relatively limited and systematic model comparison was lacking. Park et al. and Gould et al. used ML to predict 90-day and 30-day readmission after total joint arthroplasty (TJA) or total knee arthroplasty (TKA), emphasizing the need to evaluate model transferability across different centers and data types. In contrast, the present study systematically compared 11 base algorithms and a stacking ensemble model in the same dataset and evaluated performance using both ROC-AUC and PRAUC, thereby providing a more comprehensive assessment of identification ability under imbalanced outcomes (6, 15–18). Recent externally validated and registry-based studies also demonstrate that model performance varies across institutions and patient subgroups (12, 13).

Notably, although LASSO and stacking achieved higher test-set ROC-AUC values, their PRAUC values were lower than that of XGBoost. Given the low outcome-event rate in this study, PRAUC provides important information for evaluating the ability to identify positive events under class imbalance. We therefore selected XGBoost for subsequent analyses because it showed a relatively higher PRAUC while maintaining a high ROC-AUC. Nevertheless, the test-set PRAUC of XGBoost was 0.5280, indicating that positive-event identification remained moderate. In addition, the study was limited to single-center internal validation, and some tree-based models showed obvious overfitting. Therefore, the present findings support XGBoost as a supportive risk-stratification tool with predictive potential, but not as a fully validated model that can independently guide clinical decision-making. This interpretation is consistent with recent calls to consider PR-AUC, decision curves, and other multidimensional metrics when evaluating models for imbalanced medical data (18).

A distinctive feature of this study is the systematic inclusion of perioperative temperature-management and rewarming-related variables. We found that lower PACU temperature and shorter warming duration were associated with increased readmission risk. Previous studies have shown that inadvertent perioperative hypothermia is associated with coagulation dysfunction, increased blood loss, transfusion, delayed anesthetic recovery, myocardial ischemia, surgical-site infection, and other adverse outcomes. The incidence of hypothermia in major orthopedic surgery may reach 20–30%. Some evidence suggests that hypothermia in patients with hip fracture is associated with increased 30-day readmission and mortality. Although recent studies in joint arthroplasty have reported inconsistent findings regarding the association between hypothermia and 90-day outcomes, our results support the importance of strengthened temperature monitoring and rewarming during the PACU period in patients undergoing hip surgery (19–21). Randomized and systematic-review evidence supports active warming to reduce perioperative hypothermia in arthroplasty, and hypothermia in hip-fracture populations has been associated with worse short-term outcomes (22–24).

Low preoperative albumin ranked first in the SHAP analysis and showed an inverse association with readmission risk, indicating that lower albumin levels were associated with higher risk. This finding is consistent with previous evidence linking poor nutritional status to infection, delayed wound healing, and increased readmission risk. It also highlights the importance of nutritional risk screening and optimization within perioperative enhanced recovery after surgery (ERAS) pathways, particularly in older and multimorbid patients undergoing hip surgery (25). Contemporary reviews and health-economic studies further identify malnutrition as a modifiable perioperative risk marker associated with greater complications and resource use after total joint arthroplasty (26, 27).

From an application perspective, the XGBoost model may provide individualized estimates of readmission risk before surgery or during the early postoperative period. Preoperatively, age, ASA grade, comorbidities, and nutritional status could be used to identify high-risk patients for intensified perioperative management and post-discharge follow-up. Intraoperatively and in the PACU, real-time temperature and hemodynamic information could be used to dynamically update risk estimates and support decisions regarding rewarming strategies, transfusion, and analgesic management (28).

Combined with DCA and interpretability results, the model could be embedded in the hospital EMR system or perioperative information platform in future work to develop a clinical decision support system (CDSS) for patients undergoing hip surgery. Specifically, initial risk stratification could be performed before surgery using baseline information such as age, nutritional status, and heart failure. Risk estimates could then be dynamically updated during surgery and in the PACU using temperature-management and rewarming-related variables. Before discharge, high-risk patients could automatically trigger intensified intervention recommendations, such as prolonged warming, nutritional support, optimized discharge planning, and strengthened follow-up, whereas low-risk patients could receive simplified management while maintaining safety and improving resource allocation. This prediction-warning-intervention pathway may improve refined perioperative management and provide a basis for developing localized intelligent decision-support tools for orthopedic care (16).

Risk assessment after hip surgery should also extend beyond readmission. Older arthroplasty and hip-fracture patients are vulnerable to postoperative delirium and early postoperative neurocognitive disorders, which may delay recovery and complicate discharge planning (29–32). Recent systematic reviews and explainable machine-learning studies further show that delirium risk prediction is an active but methodologically heterogeneous field (33, 34). Future multidimensional perioperative models should evaluate whether shared predictors can support coordinated prediction of readmission and neurologic complications without diluting outcome-specific calibration.

This study has several limitations. First, it was a single-center retrospective study with a relatively limited sample size and number of outcome events. Only 31 of 720 patients experienced 30-day readmission, which may affect model-training stability, feature-selection consistency, and the ability to detect variables with weaker effects. Second, the study captured only readmissions to the same hospital within 30 days and could not identify readmissions to other hospitals; therefore, the true readmission rate and external generalizability of the model require further verification. Third, although preprocessing was restricted to the training set and an internal test set was used to reduce bias, residual confounding and distributional shifts inherent in retrospective data may still have affected model performance. Finally, the present results are based mainly on internal validation; future larger-sample, temporal-validation, and multicenter prospective studies are needed to further evaluate model robustness, transportability, and updating strategies. In addition, potentially important unmeasured clinical and health-system factors, including surgeon and team experience, the quality of discharge planning, rehabilitation and caregiver support, access to post-discharge care, and local readmission practices, were not captured and may have confounded the observed associations. Because the test set was sampled from the same institution and calendar period as the training set, external validation in geographically and operationally distinct institutions, followed by temporal validation in a later local cohort, is required before clinical deployment or model updating (7, 8, 12).

## 5 Conclusion

In this single-center cohort of 720 patients undergoing hip-related surgery, we developed and internally validated an XGBoost-based model for predicting 30-day readmission. The model showed good discrimination in the independent test set and demonstrated a degree of ability to identify positive events and provide clinical net benefit under class imbalance. SHAP analysis indicated that low preoperative albumin, heart failure, older age, low PACU temperature, and insufficient warming duration were key contributors to readmission risk. Overall, the model is currently more appropriate as a supportive tool for risk stratification and perioperative management reference, and its clinical utility should be further validated and optimized in larger, multicenter, and prospective studies. The model should not be used for independent clinical decisions until external and temporal validation demonstrate stable discrimination and calibration.

## Data Availability

The original contributions presented in the study are included in the article/Supplementary material. Further inquiries can be directed to the corresponding authors.
