# Supplementary material for: Construction and internal validation of a machine learning model for predicting 30-day readmission after hip surgery
Source: Front Med (Lausanne). 2026 Aug 27;13:1872049. doi: 10.3389/fmed.2026.1872049 (PMC13558412; doi:10.3389/fmed.2026.1872049)
Supplement: Supplementary file 1 [file Table_1.docx]

**Supplementary Table S1. Hyperparameter search spaces and final selected parameters**

| **Model** | **Hyperparameter** | **Search range** | **Search strategy** | **Final selected parameter** |
| --- | --- | --- | --- | --- |
| DT | tree_depth | 3–7 | Random grid, 20 combinations | 3 |
| DT | min_n | 5–10 | Random grid, 20 combinations | 10 |
| DT | cost_complexity | 10^-6^–10^-3^ | Random grid, 20 combinations | 0.000888 |
| RF | mtry | 2–10 | Random grid, 20 combinations | 3 |
| RF | trees | 200–500 | Random grid, 20 combinations | 414 |
| RF | min_n | 20–50 | Random grid, 20 combinations | 48 |
| XGBoost | mtry | 2–8 | Random grid, 20 combinations | 5 |
| XGBoost | min_n | 5–20 | Random grid, 20 combinations | 19 |
| XGBoost | tree_depth | 1–3 | Random grid, 20 combinations | 1 |
| XGBoost | learn_rate | 10^-3^–10^-1^ | Random grid, 20 combinations | 0.0275 |
| XGBoost | loss_reduction | 10^-3^–10^0^ | Random grid, 20 combinations | 0.159 |
| XGBoost | sample_size | 0.8–1.0 | Random grid, 20 combinations | 0.907 |
| LightGBM | mtry | 2–8 | Random grid, 20 combinations | 3 |
| LightGBM | trees | 100–500 | Random grid, 20 combinations | 257 |
| LightGBM | min_n | 5–10 | Random grid, 20 combinations | 10 |
| LightGBM | tree_depth | 1–3 | Random grid, 20 combinations | 3 |
| LightGBM | learn_rate | 10^-3^–10^-1^ | Random grid, 20 combinations | 0.0924 |
| LightGBM | loss_reduction | 10^-3^–10^0^ | Random grid, 20 combinations | 0.149 |
| SVM | cost | 10^-5^–10^5^ | Random grid, 20 combinations | 4.14 |
| SVM | rbf_sigma | 10^-4^–10^-1^ | Random grid, 20 combinations | 0.0322 |
| MLP | hidden_units | 15–24 | Regular grid, 3 levels | 15 |
| MLP | penalty | 10^-3^–10^0^ | Regular grid, 3 levels | 1 |
| MLP | epochs | 50–150 | Regular grid, 3 levels | 50 |
| KNN | neighbors | 3–11 | Random grid, 20 combinations | 11 |
| KNN | weight_func | Preset candidate functions | Random grid, 20 combinations | cos |
| LASSO | penalty | 10^-5^–10^0^ | Regular grid, 20 combinations | 0.0264 |
| Ridge | penalty | 10^-5^–10^0^ | Regular grid, 20 combinations | 1 |
| Elastic Net | mixture | 0–1 | Regular grid, 5 × 20 combinations | 0 |
| Elastic Net | penalty | 10^-5^–10^0^ | Regular grid, 5 × 20 combinations | 1 |

Abbreviations: DT, decision tree; KNN, k-nearest neighbors; LASSO, least absolute shrinkage and selection operator; LightGBM, light gradient boosting machine; MLP, multilayer perceptron; RBF, radial basis function; RF, random forest; SVM, support vector machine; XGBoost, extreme gradient boosting.
